# A microCT Study of Three-Dimensional Patterns of Biomineralization in Pig Molars

**DOI:** 10.3389/fphys.2018.00071

**Published:** 2018-02-09

**Authors:** Susanna S. Sova, Leo Tjäderhane, Pasi A. Heikkilä, Jukka Jernvall

**Affiliations:** ^1^Developmental Biology Program, Institute of Biotechnology, University of Helsinki, Helsinki, Finland; ^2^Department of Geoscience and Geography, University of Helsinki, Helsinki, Finland; ^3^Department of Oral and Maxillofacial Diseases, Helsinki University Hospital, University of Helsinki, Helsinki, Finland; ^4^Institute of Dentistry and Medical Research Center Oulu, Oulu University Hospital, University of Oulu, Oulu, Finland

**Keywords:** teeth, tooth maturation, biomineralization, sus scrofa, 3D-imaging, microtomography, beam hardening artifacts

## Abstract

Domestic pig molars provide an interesting system to study the biomineralization process. The large size, thick enamel and complex crown morphology make pig molars relatively similar to human molars. However, compared to human molars, pig molars develop considerably faster. Here we use microCT to image the developing pig molars and to decipher spatial patterns of biomineralization. We used mineral grains to calibrate individual microCT-scans, which allowed an accurate measure of the electron density of the developing molars. The microCT results show that unerupted molars that are morphologically at the same stage of development, can be at markedly different stage of enamel biomineralization. Erupted molars show increased electron density, suggesting that mineralization continues in oral cavity. Yet, our comparisons show that human enamel has slightly higher electron density than pig enamel. These results support the relatively low hardness values and calcium level values that have been reported earlier in literature for pig teeth. The mineral calibration was an efficient method for the microCT-absorption models, allowing a relatively robust way to detect scanning artifacts. In conclusions, whereas thin sections remain the preferred way to analyze enamel features, such as incremental lines and crystal orientation, the microCT allows efficient and non-destructive comparisons between different teeth and species.

## Introduction

The domestic pig (*Sus scrofa domesticus*) is a plant-dominated omnivore with low-crowned, multi-cusped molars. The relatively fast development of teeth together with their large size and thick enamel makes pig teeth an interesting model for biomineralization studies. The general morphology and tissue structure of pig teeth is closer to human teeth than that of rodent teeth (Robinson et al., 1987; Mlakar et al., 2014). The large size and complex tooth morphology of pig molars, however, makes them also challenging to investigate.

We use x-ray microtomography (microCT)-imaging to document the maturation of domestic pig molars, and compare the microCT models with photomicrographs from polished thin sections of the molars. According to Kirkham et al. (1988), pig enamel is relatively immature at the time of the tooth eruption, and the surface hardness of erupted pig teeth is “astonishingly low” compared to other domestic animal teeth and human teeth (Karlström, 1931).

Computational microCT is becoming an increasingly used method in paleontological, biological and medical studies (e.g., Ortiz et al., 2012; Tafforeau et al., 2012; Schmitz et al., 2014). MicroCT is a relatively rapid and non-destructive method to image different size of specimens, and to create a precise 3D-model of specimens surface and internal structure. One of our methodological interests is to test if mineral grains can be used in microCT as internal reference for relative calibration of absorption models. Different absorption models, even of similar samples, are difficult to compare because absorption values are not absolute and are affected by several scanning variables. So-called phantoms are normally used in microCT calibration, but when working with highly mineralized samples such as tooth enamel, the electron densities of the phantoms are below the electron densities of the samples (e.g., Farah et al., 2010). Due to the large size of pig molars, individual teeth need to be scanned one at a time in most commercial microCT instruments, necessitating reliable methods to normalize each scan. Apatite single crystal is the best comparable to the hydroxylapatite of the teeth. Siderite is an iron carbonate mineral with the highest electron density of our standard samples, and quartz is a pure silicon dioxide mineral, whose electron density is comparable to less mineralized tooth material, for example dentin.

## Materials and methods

### Samples

The permanent molars were extracted from half-mandibles of approximately 6 months old healthy production pigs (breed unknown). The first molars (M1) were in occlusion, the second and third molars (M2 and M3) were not erupted. The M2 crowns were fully formed but the root formation had not started yet. The third molars (M3) were still very small relative to their final size. The unerupted teeth were extracted in their dental sacs and all tooth samples were stored in 70% ethanol at 4°C. All the procedures of this study involving animals were reviewed and approved by relevant Animal Welfare and Research Committees.

Human third molars were extracted as part of a normal dental treatment and used for the study with the patients' informed consent. Immediately after the extraction, the teeth were stored in phosphate-buffered saline—containing 0.02% sodium azide, and transferred into 70% ethanol within 1 week after the extraction.

### microCT

The microCT measurements of the pig molars were performed at the Laboratory of X-ray Microtomography at the Department of Physics, University of Helsinki, Finland. The imaging set-up is a custom-built microtomography system, which is manufactured by phoenix|X-ray System and Service GmbH (General Electric, Wunstorf, Germany). The x-ray source is a fixed-target transmission type x-ray tube with a tungsten filament cathode and a tungsten transmission anode, target that is fused onto a 400 μm thick beryllium window.

The following parameters were used in the study: source voltage of 120 kV, source current of 170 μA, imaging voxel size was 10 to 24 μm (or 2 to 4 enamel rods) 5 mm Al-filter, projection images were taken over rotation step of (360°/1200 rotation, exposure time 500 to 750 ms, and frame averaging of 8). Tomography reconstructions were obtained using the program Datos |X by phoenix|X-ray System and Service GmbH (General Electric). Human and pig molars comparison was obtained using SkyScan 1272 desktop micro-CT system, Bruker microCT N.V., Kontich, Belgium. All absorption models were down-sampled to 44 μm voxel size for the analyzes.

**Figure 1:**
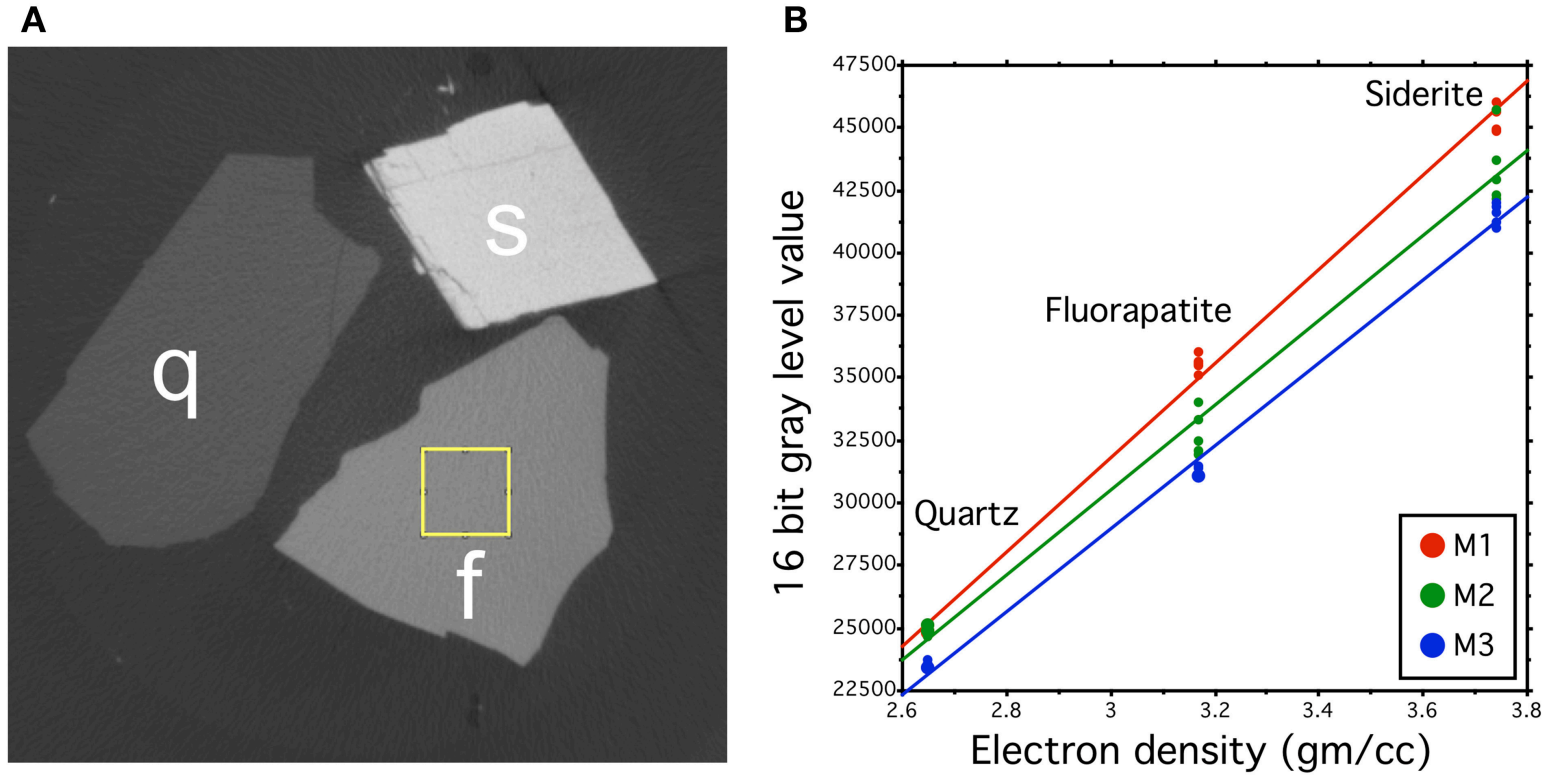
Calibration method for the microCT absorption models. **(A)** Cross-sections of the mineral standards quartz (Q), siderite (S), and fluorapatite (F) in one microCT model, showing different gray values that correlate with the electron density of the material. The rectangle in F represents one area, from where the gray value of the grain was measured. **(B)** Comparison of the calibration lines of three different samples (M1, M2, M3). The slopes of each calibration line are relatively equal, but slightly spreading toward higher electron density values. Each point represents one gray value measurement of the mineral standard and its theoretical electron density value.

For the microCT-imaging, we selected three single-crystal mineral grains to be used as internal reference samples: fluorapatite (Ca_5_(PO_4_)_3_F), quartz (SiO_2_) and siderite (FeCO_3_). Each molar was scanned together with three mineral grains that were used as an internal reference for relative calibration for the microCT absorption models. The gray-scale values of the mineral grains in the absorption models were measured from a 1 mm^2^ homogenous area at the center of each mineral grain (Figure 1A). The gray-scale measurement was repeated five times from non-overlapping areas within each mineral grain and the results were averaged. The gray-scale values of each mineral were correlated with its (theoretical) electron density values (Barthelmy, 2014) to produce a calibration line for each model. The calibrated values were used to convert the gray-scale values of each model to relative electron density values (RED). Comparison of the calibration lines between the reconstructions reveals the initial gray-scale value discrepancies between the scans. The slopes of each calibration line are relatively equal, however, showing that the electron density range that was covered by the mineral reference standards, can be used to convert the gray-scale value ranges of each tooth measurement to comparable RED-values (Figure 1B). The analyses were done using Fiji (https://fiji.sc/).

### Polarized light microscopy

One cross-section from each pig molar (M1, M2, M3) was prepared for microscopic examinations. Prior to the thin section preparation, the teeth were covered with epoxy to avoid fracturing. The polished thin sections (thickness 30 μm) were studied and photographed using Leica DM2500P polarized light microscope equipped with Leica DFC450C camera.

## Results

### MicroCT absorption models of pig molars

**Figure 2:**
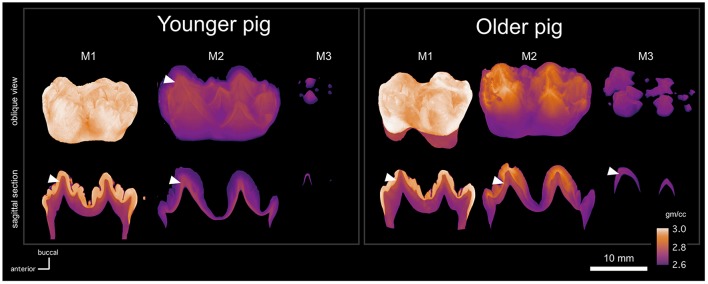
MicroCT absorption models of the molars (M1, M2, M3) of two pig individuals (younger and older), showing the shape (**upper row**) of each tooth, where the maturation stage is represented by reduced transparency, and the electron density by calibrated false color scale. Sagittal cross sections (**lower row**) of each tooth show mutually comparable electron density variation across each profile with EDJ marked with the white arrow heads.

The absorption models of the molars show small differences in development between pig individuals, here referred as the younger and the older pigs for brevity (Figure 2). The first molars of both pigs appear equal electron density (degree of mineralization), but the dentin layer is slightly thicker in the older pig individual. The second molars are morphologically similar and the enamel thickness is roughly equal, but the enamel maturation is more advanced in older pig than in the younger one. In the older pig M2, the electron density of enamel is higher than that of dentin in the cusps, but not in the neck. In the younger pig, the electron density of enamel is relatively uniform, but distinctly lower than that of the dentin. As a consequence, the morphology of the enamel-dentin-junction (EDJ) is distinguishable through the enamel in the absorption model (Figure 2).

Robinson et al. (1987) described enamel maturation using a partially visual classification scheme. Stages 1 and 2 enamel is translucent whereas stage 3 enamel is white and opaque. Stage 4 enamel is hard and translucent. Compared to stage 1, stage 2 enamel cracks vertically when let to dry. These stages correspond to chemical and histological changes related to maturation (Robinson and Kirkham, 1984; Robinson et al., 1987,1988), and some of the stages can also be distinguished from our absorption models. Based on our visual inspection of the molars, the first molars were at the stage 4 and the third molars were at the stage 1, whereas the second molars showed differences. The whole crown of the younger pig M2 was at stage 1, whereas the top 57% (of 14 mm total) of the older pig crown was at stage 3. In our absorption models, the stage 1 and 2 enamel correspond to electron densities that are lower than that of dentin, and the stage 3 enamel of the older pig M2 shows higher than dentine electron density values (Figure 2).

**Figure 3:**
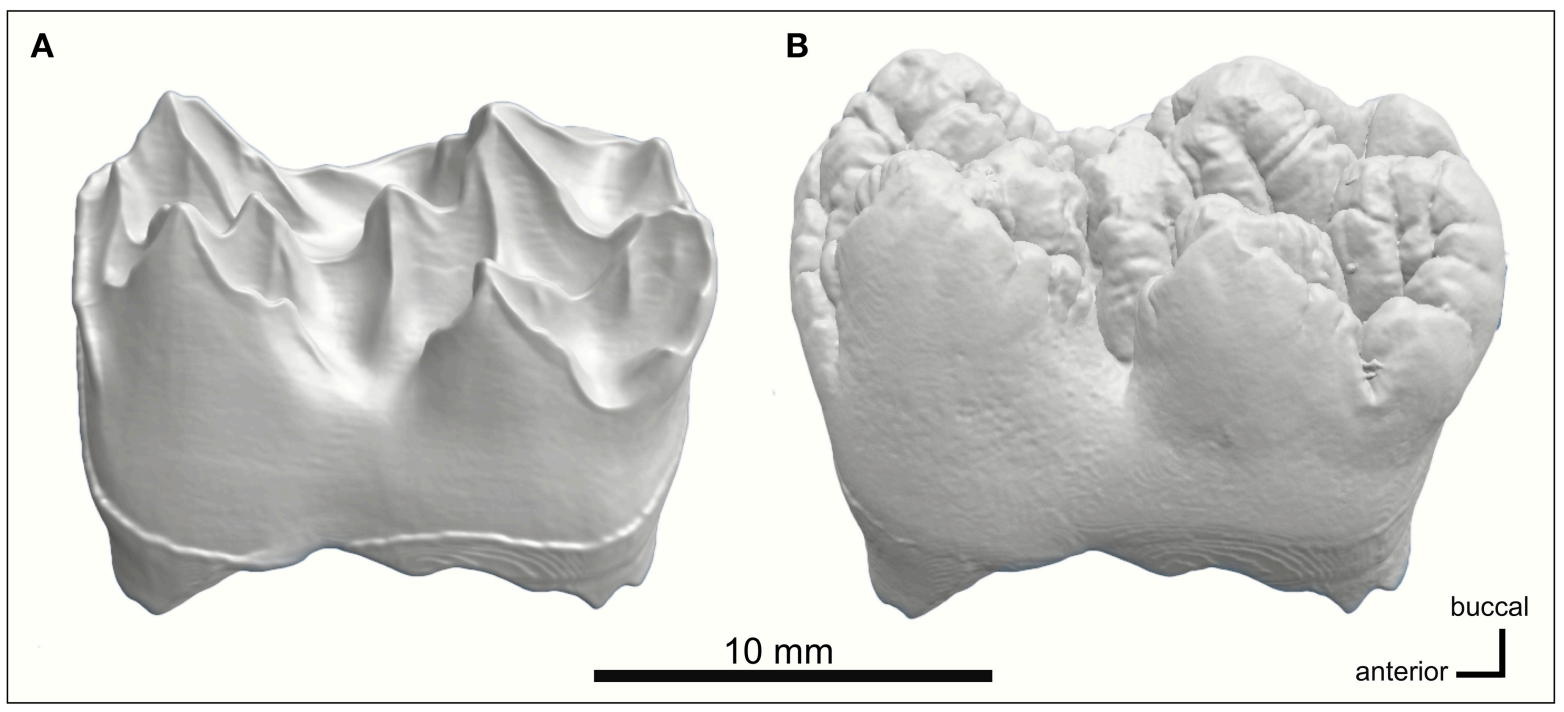
MicroCT models of **(A)** EDJ morphology and **(B)** tooth surface of the younger pig M1. The enamel is digitally removed using its electron density threshold. Enamel surface shows rounded, convex cusps with narrow intervening fissures, whereas the EDJ is characterized by sharp cusp tips and ridges with concave valleys in between them.

The pig M2 reveal distinctly different morphologies between EDJ and enamel surface: The EDJ has sharp cusp tips and ridges with concave valleys between them, but the enamel surface has convex cusps with narrow fissures in between the cusps. A similar EDJ-morphology is visible in the M1 absorption model, when the enamel layer is digitally removed (Figures 3A,B). As in M1, the dentin of M2 is thicker in the older pig than in the younger pig. In the third molars, the mineralization has only begun at the tips of the principal cusps. The order of development from the anterior to posterior parts of the molars, is well visible in the younger M3 in which the mineralized tips of talonid cusps are barely visible and still close to each other, indicating that the growth of the valley separating the cusps has not yet been completed (Figure 2). With the older pig M3, more cusps are distinguishable than in the younger pig M3 and the valleys between the principal cusps are larger.

**Figure 4:**
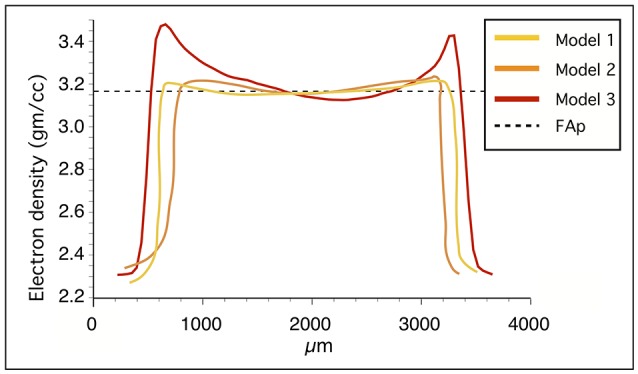
Electron density profiles of the fluorapatite standard in three microCT absorption models. Models 1 and 2 have consistent values whereas model 3 shows higher electron density at the sample surface due to beam-hardening.

Because mineral grains used for calibration have reasonably uniform electron densities, we discovered that an additional benefit of the minerals is their suitability for detecting beam-hardening artifacts from the microCT models (Figure 4). Beam hardening makes the sample appear artificially denser at or near its surface, and less dense in its central parts (Boas and Fleischmann, 2012). The x-rays produced in laboratory x-ray sources are polychromatic and have mixed x-ray photon energy. As this mixed beam of x-rays passes through the sample, the lower energy x-rays will be attenuated more rapidly than the higher energy x-rays, resulting in brighter edges if beam attenuation is considered linear. The hardening effects can be reduced during scanning or with specific algorithms that are used during reconstruction. However, these artifacts can be particularly challenging to recognize in tooth samples: teeth usually have a thin layer of highly mineralized enamel on the surface that can be difficult to distinguish from the hardening artifact as thick as the whole enamel layer, especially in small teeth with thin enamel. In our scans, the relatively large apatite crystal shows a flat electron density profile in microCT models without hardening artifacts, but a concave profile in a microCT model with hardening artifacts (Figure 4).

### Comparison of the microCT absorption models and thin sections

**Figure 5:**
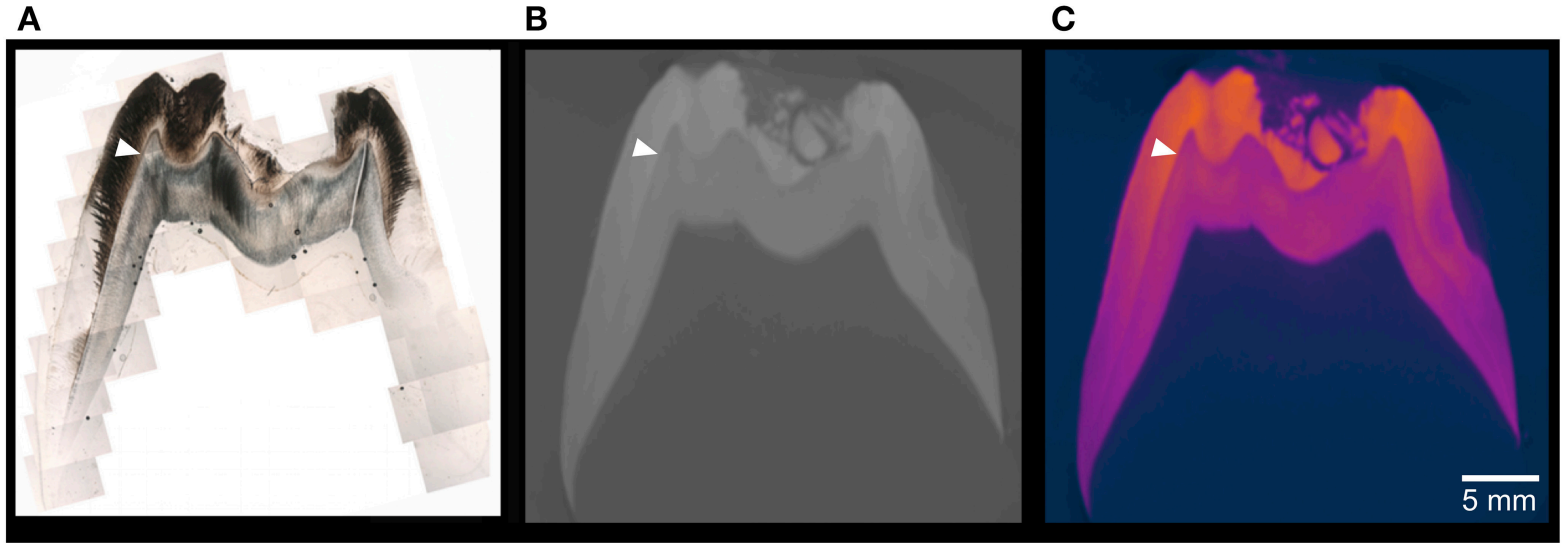
Cross sections of the older pig M2. **(A)** Combined photomicrograph of a thin section and **(B)** Cross section of the microCT model in electron-density-calibrated in gray scale, and false colors **(C)**. The ultrastructure of enamel and dentin under polarized light microscope is superior to the microCT model and false colors aid the detection of differences in electron density.

**Figure 6:**
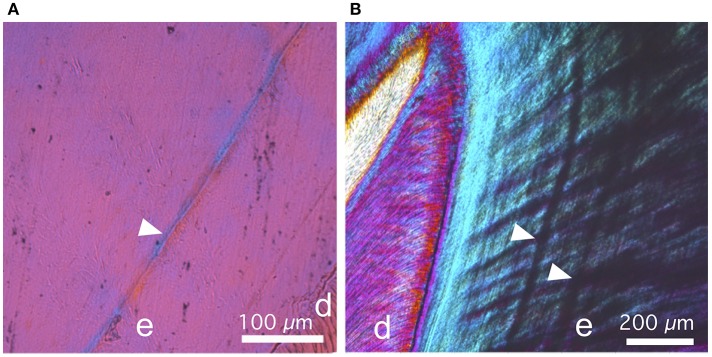
Details of thin sections of the older stage pig, photographed using cross-polarized light and gypsum accessory plate. **(A)** In M1, the neonatal line (arrow head) is optically more coherent than the surrounding enamel, as evidenced by the stronger blue and yellow interference colors. **(B)** In M2, the most pronounced incremental lines appear opaque, and therefore distinct from the neonatal line.

Physical thin sections of teeth and virtual sections made from the microCT absorption models show similar patterns of maturation (Figures 5A–C). However, thin sections also show details that are not visible in microCT absorption models. For example, the cross-polarized light and additional gypsum plate allow the direct observation of the principal crystal orientation (Figures 6A,B). In the M1 thin section, the neonatal line is optically more coherent than the surrounding enamel. Contrary to the neonatal line, the most pronounced incremental lines observed in M2 are opaque.

### Electron density comparison of pig and human molars

**Figure 7:**
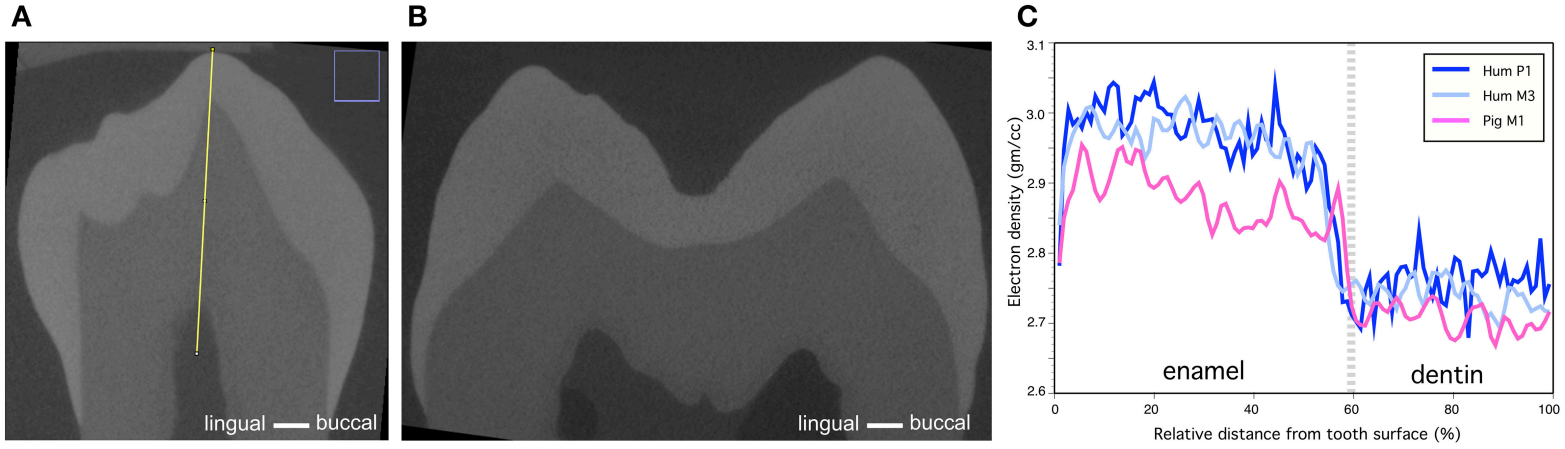
Comparison of human and pig molar electron density. **(A)** Cross-section of human P1 (Yellow line denotes the line of measurements) **(B)** Cross-section of human M3. **(C)** Comparison of electron density of human and older stage pig M1 show that human molar has slightly higher electron density in enamel and the difference is negligible in dentin.

Comparison of the calibrated microCT models of pig M1, human P1 and M3 show that human enamel has slightly higher electron density values than pig enamel (Figures 7A–C). The differences in the electron density of dentin are small.

## Discussion

The microCT absorption models provide a good overall view of the developing pig molars. 3D-models show the timeline of tooth maturation in two directions: in anterior-posterior axis, and from the tooth cusps toward the tooth neck. MicroCT-models also show how the maturation of enamel takes longer than that of the dentin. The transition point when the electron density of enamel exceeds that of dentin, appears to correspond to the transition from stage 1-2 enamel to stage 3 enamel in the classification scheme used by Robinson et al. (1987). This suggests that microCT could potential be used for non-invasive classification of enamel maturation.

The mineral calibration of the microCT model proved to be an efficient method to re-scale the electron density values to comparable semi-quantitative scale. In addition, mineral grains are useful for the detection of beam hardening artifacts when the properties of the investigated samples are unknown. The electron density of fluorapatite is higher than that of hydroxylapatite that is again slightly higher than the electron density of enamel. Accordingly, it could be sufficient to use fluorapatite as the highest electron density standard in bio-apatite research instead of siderite. In addition to fluorapatite and quartz, diamond could be utilized as the lowest electron density reference. Diamond and quartz are also available as pure synthetic phases. An obvious future extension of our calibration method is to compare the absorption of the minerals to hydroxyapatite phantoms (Schweizer et al., 2007).

Whereas the microstructure of tooth and the orientation of crystals (e. g. enamel rods and their decussation) are not visible in absorption models, they are detectable in thin sections. However, 3D detection of enamel microstructure is possible using synchrotron x-ray imaging (e.g., Tafforeau et al., 2012). The polarized light microscopy observations showed the different appearance of the neonatal line and other incremental lines. The higher optical coherence indicates more parallel crystal orientation in neonatal line than elsewhere in enamel, and could indicate that the crystal nucleation was arrested at the time of the birth.

Kirkham et al. (1988) studied the mineral content of pig enamel. Their results showed that the mineral content of pig enamel at eruption is 50–60% per volume, which is near or equal to the mineral content of human dentin and considerably lower than the mineral content of human enamel (Jenkins, 1976). In the pig molars that we studied, the electron density of enamel is higher than that of dentin before the tooth eruption (Figure 2). According to our microCT models, the electron density of human enamel is slightly higher than that of pig enamel, whereas the differences in the electron densities of dentin are negligible (Figure 7C). Kirkham et al. (1988) state that the characteristics of mature mammal enamel could be achieved in pig teeth considerably later after tooth eruption. In our samples, the first molars of pigs had been in oral cavity approximately for 2 months whereas the human M3s were extracted from adults. In human teeth, the enamel surface still hardens in oral cavity at least for 2–3 years (Lynch, 2013).

One possible reason for the low electron density values of erupting pig molars could be the relatively fast development of pig teeth. Pig M1 develops from the beginning of the bud stage to the eruption in approximately 6.5 months whereas with human this takes 6 years (Kraus and Jordan, 1965; Tonge and McCance, 1973; Berkovitz et al., 2009; Wang et al., 2014). In other words, pig tooth development is almost 10 times faster than human tooth development, even though the pig M1 size is almost twice that of human M1. Studies on rat incisor eruption suggest that incisors compensate for artificially accelerated eruption by having wider zone of secreting ameloblasts (Robinson et al., 1988). Whereas, it is not known how molars that develop rapidly regulate their development, the low electron density values of erupting pig molars could indicate the maturation stage as a limiting factor. Another consideration is that in most ungulates, the secondary morphology of molars is functionally more important than the primary morphology, and immature enamel in erupting molars may help to quickly acquire the functional morphology. In the domestic pig, both primary and secondary morphologies are functional. Nevertheless, despite the differences between pig and human molars, they have comparable tooth replacement patterns and bunodont crown features such as thick enamel with deep furrows between cusps.

Finally, Limeback et al. (1992) reported difficulties in detecting the effects of Vitamin-D deficiency in pig enamel radiographically, and it remains to be tested whether mineralization defects can be detected in pigs using calibrated microCTs. The difficulty in detecting vitamin D defects may also be connected to the fast tooth development in pigs. With the increasing efficiency of genome editing of large animals (Whitelaw et al., 2016), it is conceivable that new pig-models suitable for dental studies will be available in the future.

In conclusion, microCT is a valuable starting point for the traditional destructive methods used in the study of biomineralization, but does not substitute for them. Thin sections still provide valuable information about the tooth structure. The optical differences between neonatal line and other incremental lines were clear under polarized light microscope, warranting further study. Mineral grains as internal standards can be used to calibrate microCT scans, allowing the detection of scanning artifacts and comparisons between developmental stages and species. Our results show that the fast developing pig molars complete their enamel matrix deposition over the whole crown prior to the maturation. Considering that pig enamel is very thick, the combination of microCT imaging and pig molars is a useful model system in the study of biomineralization.

## Author contributions

SS, PH, and JJ designed the project. SS acquired the data and performed the imaging and analyses. LT provided material, observations and scientific interpretations. SS wrote the initial manuscript and all authors discussed the results and provided input on the manuscript.

### Conflict of interest statement

The authors declare that the research was conducted in the absence of any commercial or financial relationships that could be construed as a potential conflict of interest.

## References

[B1] Barthelmy D. (2014). Mineralogy Database. Available online at: http://www.webmineral.com/

[B2] Berkovitz B. K. B. Holland G. R. Moxham B. J. (2009). Oral anatomy, Histology and Embryology, 4th Edn. Edinburgh: Mosby/Elsevier.

[B3] Boas F. E. Fleischmann D. (2012). CT artifacts: causes and reduction techniques. Imaging Med. 4, 229–240. 10.2217/iim.12.13

[B4] Farah R. A. Swain M. V. Drummond B. K. Cook R. Atieh M. (2010). Mineral density of hypomineralized enamel. J. Dent. 38, 50–58. 10.1016/j.jdent.2009.09.002 19737596

[B5] Jenkins N. G. (1976). The Physiology and Biochemistry of the Mouth, 4th Edn, (Oxford: Blackwell Scientific Publications), 54–112.

[B6] Karlström S. (1931). Physical, Physiological and Pathological Studies of Dental Enamelwith Special References to the Question of its Vitality. Stockholm: A. B. Fahlcrantz' Boktryckeri.

[B7] Kirkham J. Robinson C. Weatherell J. A. Richards A. Fejerskov O. Josephsen K. (1988). Maturation in developing permanent porcine enamel. J. Dent. Res. 67, 1156–1160. 10.1177/00220345880670090301 3165998

[B8] Kraus B. S. Jordan R. E. (1965). The Human Dentition Before Birth Philadelphia, PA: Lea and Febiger.

[B9] Limeback H. Schlumbohm C. Sen A. Nikiforuk G. (1992). The effects of hypocalcemia hypophosphatemia on porcine bone and dental hard tissues in an inherited form of type-1 pseudo-vitamin-d deficiency rickets. J. Dent. Res. 71, 346–352. 10.1177/00220345920710020101 1556293

[B10] Lynch R. J. M. (2013). The primary and mixed dentition, post eruptive enamel maturation and dental caries: review. Int. Dent. J. 63, 3–13. 10.1111/idj.12076 24283279 PMC9375027

[B11] Mlakar N. Pavlica Z. Petelin M. Štrancar J. Zrimšek P. (2014). Animal and human dentin microstructure and elemental composition. Cent. Eur. J. Med. 9, 468–476. 10.2478/s11536-013-0295-x

[B12] Ortiz A. Skinner M. M. Baileya S. E. Hublin J.-J. (2012). Carabelli's trait revisited: an examination of mesiolingual features at the enamel-dentin junction and enamel surface of Pan and Homo sapiens upper molars. J. Hum. Evol. 63, 586–596. 10.1016/j.jhevol.2012.06.003 22889682

[B13] Robinson C. Kirkham J. (1984). Is the rat incisor typical. INSERM 125, 377–388.

[B14] Robinson C. Kirkham J. Nutman C. A. (1988). Relationship between enamel formation and eruption rate in rat mandibular incisors. Cell Tissue Res. 254, 655–658. 3233657 10.1007/BF00226516

[B15] Robinson C. Kirkham J. Weatherell J. A. Richards A. Josepsen K. Fejerskov O. (1987). Developmental stages in permanent porcine enamel. Acta Anatomica 128, 1–10. 10.1159/000146306 3825482

[B16] Schmitz J. E. Teepe J. D. Hu Y. Smith C. E. Fajardo R. J. Chun Y.-H. P. (2014). Estimating mineral changes in enamel formation by ashing BSE and MicroCT. J. Dent. Res. 20, 1–7. 10.1177/0022034513520548 PMC3929980 24470541

[B17] Schweizer S. Hattendorf B. Schneider P. Aeschlimann B. Gauckler L. Müller R.. (2007). Preparation and characterization of calibration standards for bone density determination by micro-computed tomography. Analyst 132, 1040–1045. 10.1039/b703220j 17893808

[B18] Tafforeau P. Zermeno J. Smith T. (2012). Tracking cellular-level enamel growth and structure in 4D with synchrotron imaging. J. Hum. Evol. 62, 424–428. 10.1016/j.jhevol.2012.01.001 22304852

[B19] Tonge C. H. McCance R. A. (1973). Normal development of the jaws and teeth in pigs, and the delay and malocclusion produced by calorie defiencies. J. Anat. 155, 1–22. PMC1271522 4199500

[B20] Wang F. Xiao J. Cong W. Li A. Song T. Wei F.. (2014). Morphology and chronology of diphyodont dentition in miniature pigs, Sus Scrofa. Oral Disease 20, 367–379. 10.1111/odi.12126 23679230

[B21] Whitelaw C. B. Sheets T. P. Lillico S. G. Telugu B. P. (2016). Engineering large animal models of human disease. J. Pathol. 238, 247–256. 10.1002/path.4648 26414877 PMC4737318

